# Supplementary material for: Tuning colloidal quantum dot band edge positions through solution-phase surface chemistry modification
Source: Nat Commun. 2017 May 16;8:15257. doi: 10.1038/ncomms15257 (PMC5440806; doi:10.1038/ncomms15257)
Supplement: Supplementary Information — Supplementary Figures, Supplementary Tables, Supplementary Notes and Supplementary References. [file ncomms15257-s1.pdf]

Supplementary Figures

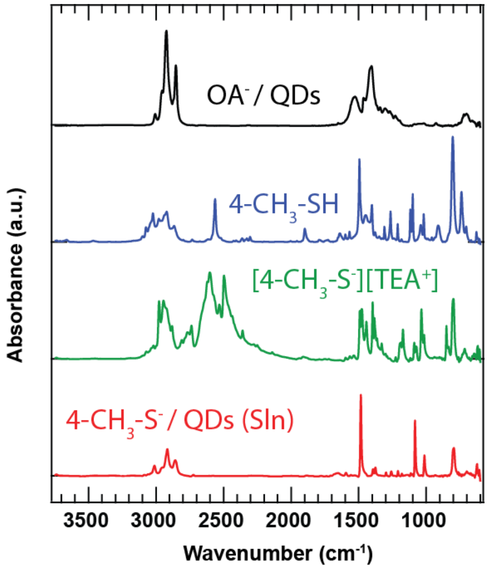

Supplementary Figure 1. FT-IR absorbance spectra of ligands and ligand/QD complexes associated with the 4-CH_3-SH/4-CH_3-S^- solution-phase ligand exchange. The red trace shows the PbS 4-CH_3-S^-/QD spectrum after three cycles of PCR purification.

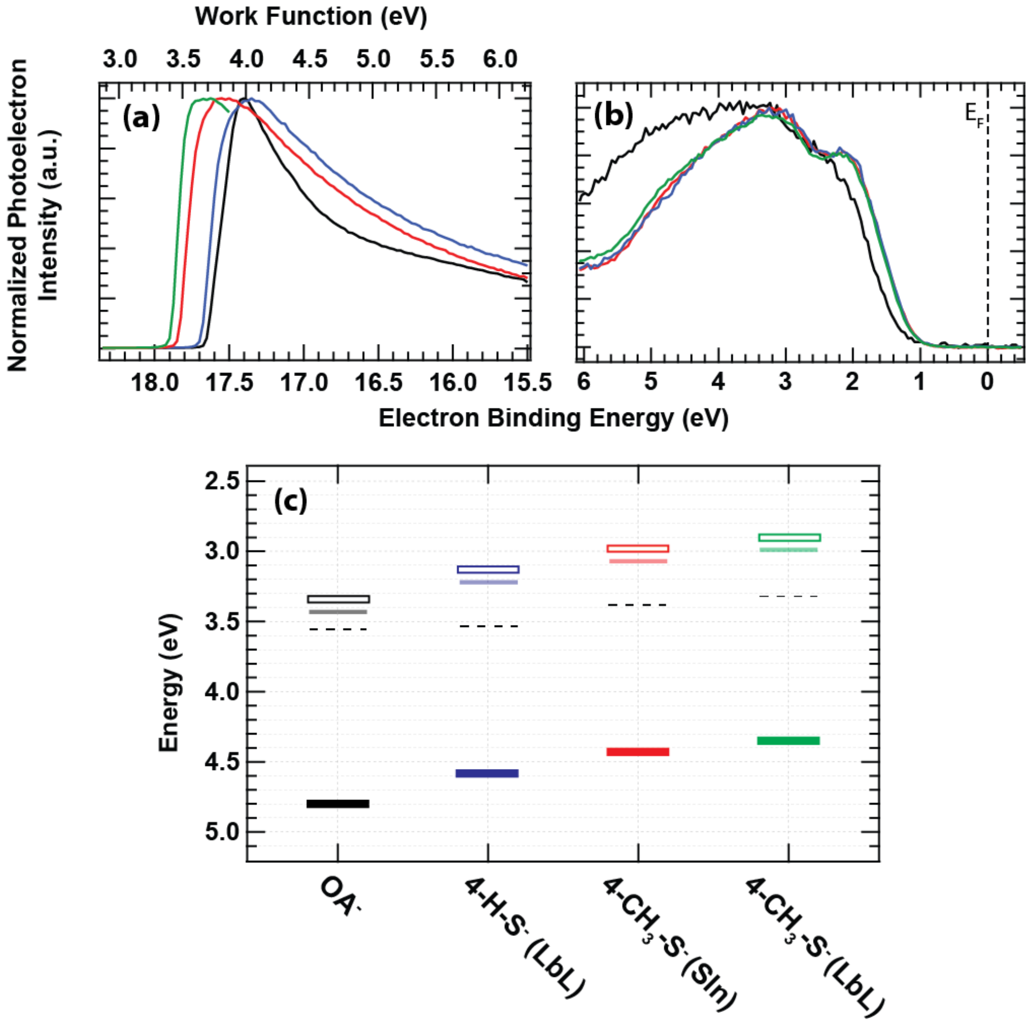

Supplementary Figure 2. XPS data used to determine the ionization energy and work function of PbS 4-R-S$^-$/QD films (a) Photoelectron cutoff region of XPS spectra used to determine ligand/QD film work function. (b) Valence band edge region of XPS spectra used to extract the ligand/QD film valence band maximum with respect to the Fermi energy. Dashed black line represents the instrument equilibrated Fermi energy at an electron binding energy of 0 eV. Color coding can be found in SupplementaryTable 3. (c) XPS measurements of films fabricated from OA$^-$ and 4-R-S$^-$ terminated 3.2 nm PbS QDs deposited using solution-phase single step (Sln) or layer-by-layer (LbL) techniques; ionization energy (solid rectangles) and work function (dashed black lines. The conduction band minimum with respect to vacuum (open rectangles) is determined from $(E_{\mathrm{F}} - E_{\mathrm{VBM}})$ – (the optical gap + exciton binding energy), where the shaded lines are a result of the optical gap only.

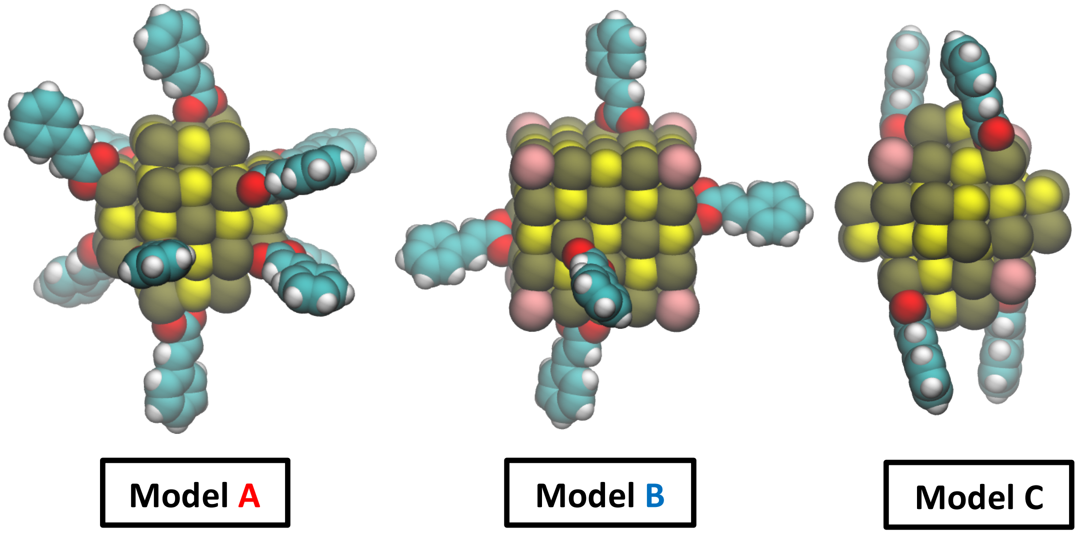

Supplementary Figure 3. Ball-and-stick structural models of representative 4-H-CA$^-$ capped PbS QDs. Red/cyan/white/pink/yellow/tan colors represent oxygen/carbon/hydrogen/iodine/sulfur/lead atoms, respectively.

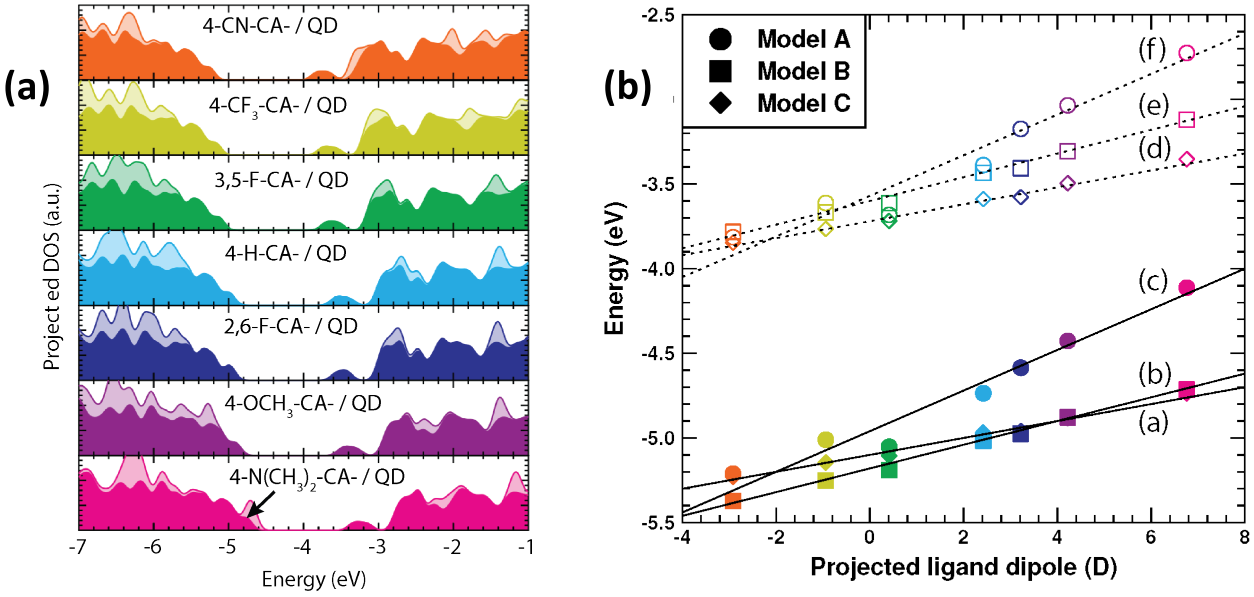

Supplementary Figure 4. R-CA$^-$/QD complex band edge calculations. (a) Projected density of states for Ligand/QD complex model C. Transparent (filled) areas represent the density of states projected onto atoms belonging to the ligands (QD core). In the case of 4-N$(CH_3)_2$-CA$^-$, an arrow highlights the first state in the vicinity of the band edge that is localized on the QD.(b) Filled (open) symbols represent the energies of the highest occupied (lowest unoccupied) QD states for the three QD/ligand models as a function of the projected dipole of the R-CAHs. See text for the definition of QD states based on the projected density of states. The lines are fits with formula (solid (dotted) lines represent occupied (unoccupied) states, respectively): (a) y=0.052x-5.1, (b) y=0.067x-5.18, (c) y=0.117x-4.96, (d) y=0.052x-3.72, (e) y=0.067x-3.6, (f) y=0.117x-3.57.

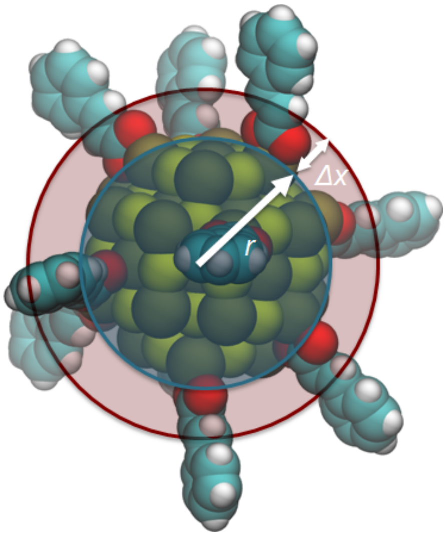

Supplementary Figure 5. Illustration of parameters used for an electrostatic ligand/QD model. In order to use the computed band edge shifts for our QD models to predict the maximum possible band edge shift in the case of experimental conditions, that is, a larger QD with more ligands, we used an electrostatic model.[1] The electrostatic model is valid under the assumption that the QD is spherical, and its radius, r, is significantly larger than the ligand/QD interface where the surface dipole layer is present, Δ*x*.

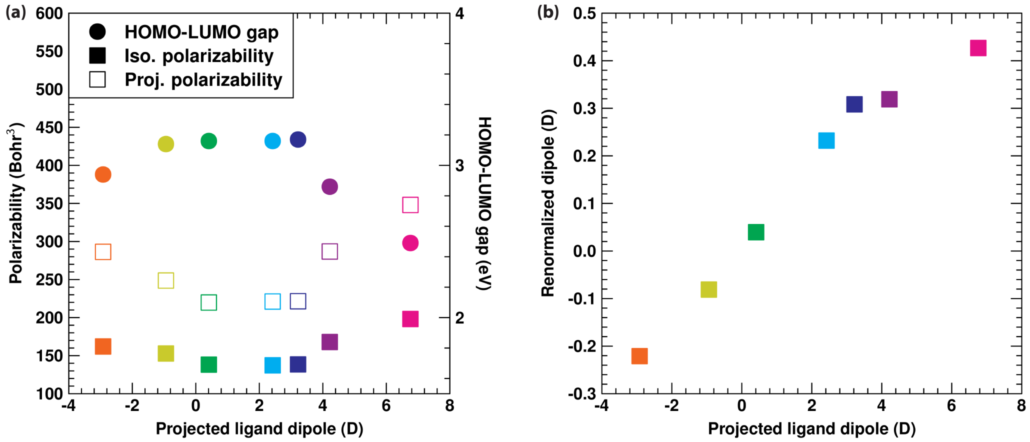

Supplementary Figure 6. Polarizability effects on ligand/QD band edges. (a) HOMO-LUMO gaps, isotropic and projected polarizabilities as a function of the projected dipole moment of the protonated ligands. (b) Projected dipoles renormalized by depolarization effects.

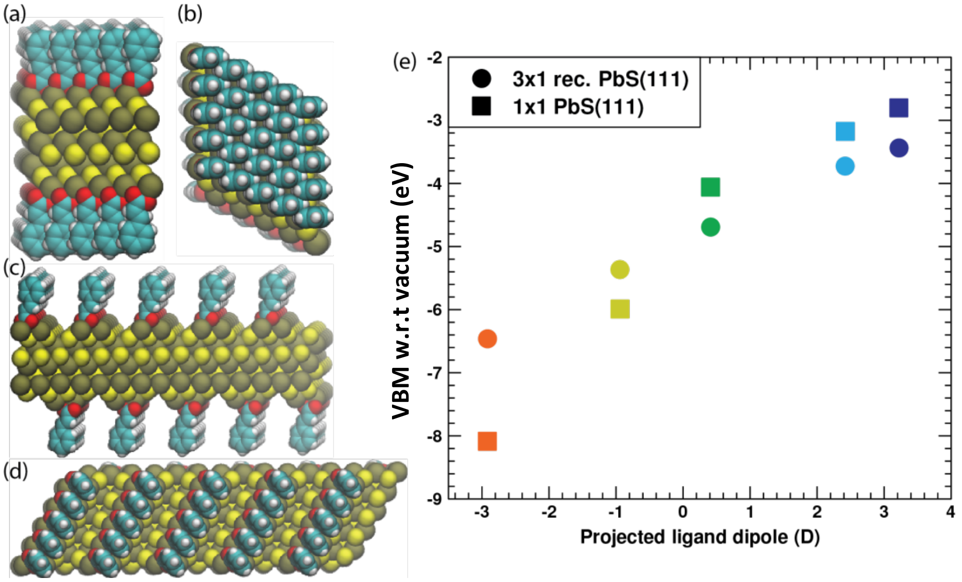

Supplementary Figure 7. Slab calculations of ligand/QD band edges (a) Side and (b) top view of 5x5 repetition of the 1x1 PbS(111) slab with full surface coverage. (c) Side and (d) top view of the 5x5 repetition of the reconstructed 3x1 PbS(111) slab. (e) Energy of the valence band maximum for the two different slab models as a function of the projected ligand dipole.

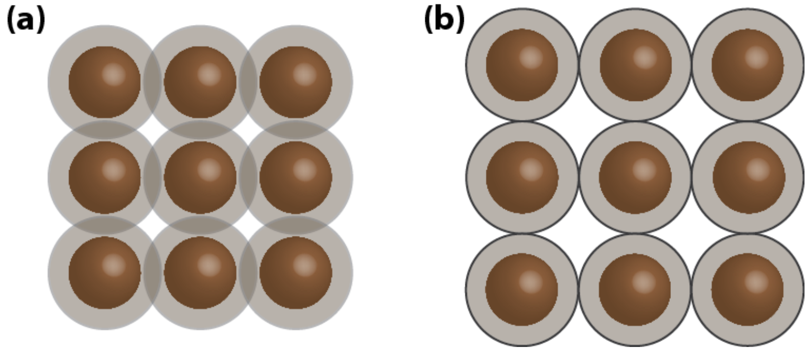

Supplementary Figure 8. Cartoon illustration of ligand shell inter-digitization. Simple two-dimensional QD monolayer models showing (a) ligand shell inter-digitization and (b) electrostatic repulsion of ligand shells preventing inter-digitization. The brown spheres represent PbS QD inorganic cores, the surrounding light gray circles represent the ligand shell, and the black circle of panel (b) represents a "hard" electrostatic perimeter preventing ligand shell inter-digitization.

(a)

(b)

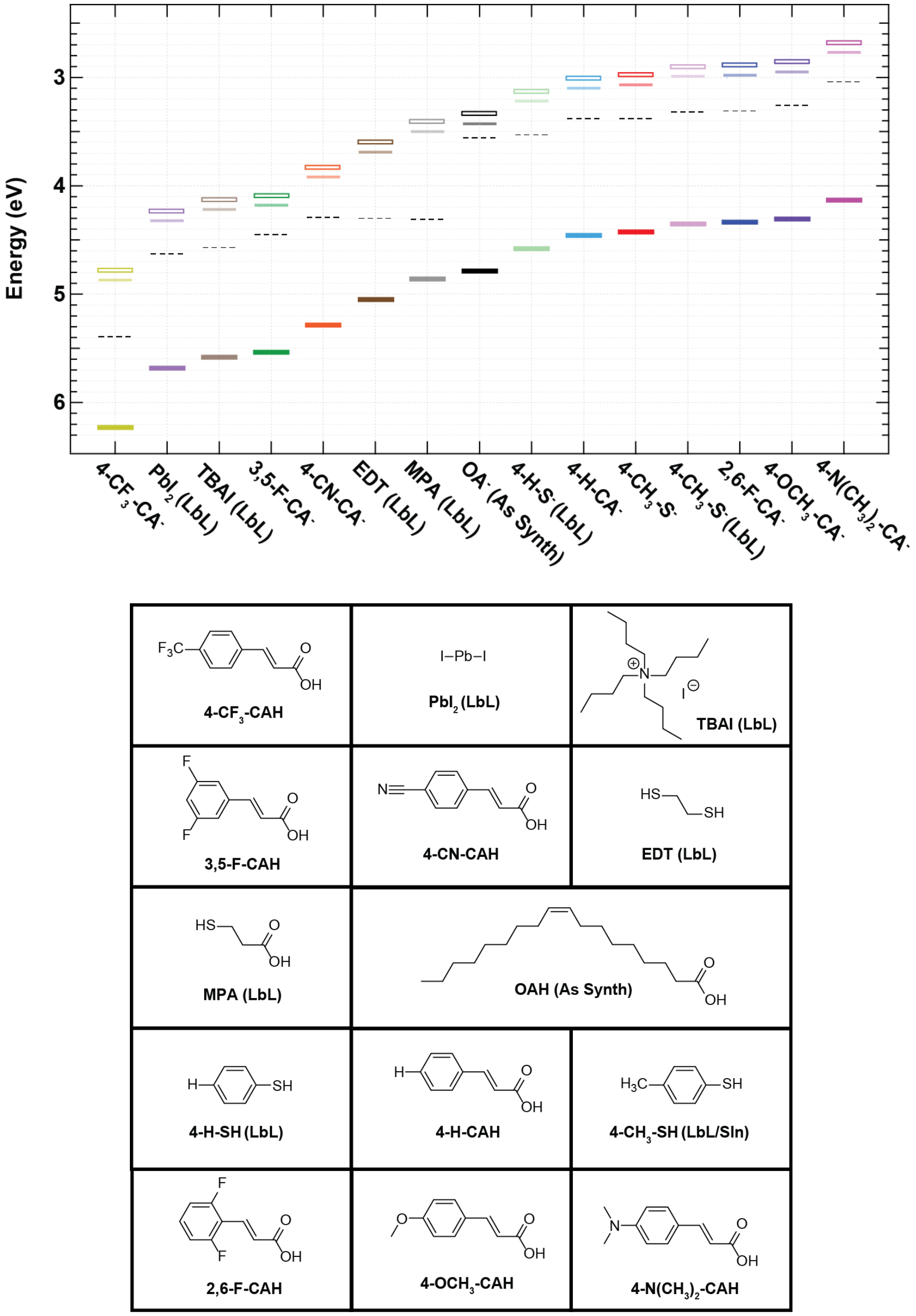

Supplementary Figure 9. R-CA$^-$/QD band edges *versus* conventional ligand/QD band edges. (a) XPS measurements of films fabricated from 3.2 nm diameter PbS QDs with various surface ligands; ionization energy (solid rectangles) and work function (dashed black lines). We also include the conduction band minimum (E_{CBM}, open rectangles) that is determined from summing the optical gap determined from absorbance measurements (opaque lines) and calculated exciton binding energy as described in the main text. Films were fabricated using either solution-phase or solid-state, layer-by-layer (LbL) exchange techniques (b) Protonated chemical structures of all of the ligands studies in this work.

Supplementary Tables

Supplementary Table 1. Ligand exchange and PCR purification details

	Ligand Solvent[1]	Antisolvent	Ligand/QD Solvent	Film Fab. Solvent
4-CN	5:1 ACN:IPA	Hexane	ACN	ACN
4-CF_3	Acetone	Hexane	MeOAc	MeOAc
3,5-F	MeOAc	Hexane	MeOAc	MeOAc
4-H	DCM	Hexane	DCM/HFIP[2]	10:1:0.01 DCM:DCB:HFIP
2,6-F	DCM	Hexane	DCM	10:1 DCM:DCB
4-OCH_3	5:1 ACN:IPA	Hexane	DCM	10:1 DCM:DCB
4-$N(CH_3)_2$	2:1 IPA:DCM	Hexane	DCM	10:1 DCM:DCB

[1]Heating and sonication were sometimes necessary to solubilize ligand.
[2]Without HFIP, exchanged QDs are insoluble. We postulate HFIP aids in breaking apart inter-QD ligand π- π interactions.
[*]Full chemical names with abbreviations in the table can be found in Methods Section of the main text.

Supplementary Table 2. XPS elemental analysis for an OA^-/QD films and R-CA^-/QD films

	Pb	S	O	C	F	N		Pb:S	O:Pb	C:Pb		E_F-E_{VBM} (eV)	Φ (eV)
OA^-	7.01	3.10	6.91	83.05	ND	ND		2.26	0.99	11.85		1.23	3.56
4-CN	10.71	5.54	11.37	67.05	ND	5.33		1.93	1.06	6.26		0.99	4.29
4-CF_3	9.69	4.26	9.55	55.07	20.79	ND		2.27	0.99	5.68		0.84	5.39
3,5-F	11.28	5.24	11.31	57.49	14.68	ND		2.15	1.00	5.10		1.09	4.45
4-H	12.15	5.79	13.46	68.59	ND	ND		2.10	1.11	5.65		1.08	3.38
2,6-F	10.84	5.22	10.03	63.80	10.10	ND		2.08	0.93	5.89		1.03	3.31
4-OCH_3	10.05	4.44	18.86	66.36	ND	ND		2.12	1.88	6.60		1.05	3.26
4-$N(CH_3)_2$	9.63	4.73	12.07	66.26	ND	5.39		2.04	1.25	6.88		1.09	3.04

[*]ND = Not Detected

Supplementary Table 3. XPS elemental analysis for an OA^-/QD films and 4-R-S^-/QD films

	Ligand Dipole (Debye)		Pb	S	O	C	N		Pb:S	O:Pb	C:Pb
4-H-S^- (LbL)	0.67		22.37	19.54	0.35	57.74	ND		1.14	0.02	2.58
4-CH_3-S^- (Sln)	1.19		16.50	14.68	2.61	66.21	ND		1.12	0.16	4.01
4-CH_3-S^- (LbL)	1.19		19.54	16.95	0.63	62.89	ND		1.15	0.03	3.22

[*]ND = Not Detected

Supplementary Table 4. The computed parameters of our electrostatic model. The first column contains the model index. The second column shows the slopes of the fitted lines shown in Supplementary Fig. 4b. The third and fourth columns represent the radius (*r*) of the QDs and the number of surface bound ligands (*N*), respectively. The fifth column is the prefactor that incorporates the specific ligand/QD core interface details (*A*). Finally, the last column shows the slope for the experimental conditions (see text for details).

	AN/r^2 (eV/D)	*r* (nm)	*N*	*A*	AN/r^2 (eV/D)*
**Model A**	0.117	0.880	10	0.0091	0.355
**Model B**	0.067	0.765	6	0.0065	0.255
**Model C**	0.052	0.770	4	0.0077	0.301

* Computed for r = 1.6 nm and N = 100

Supplementary Notes

Supplementary Note 1

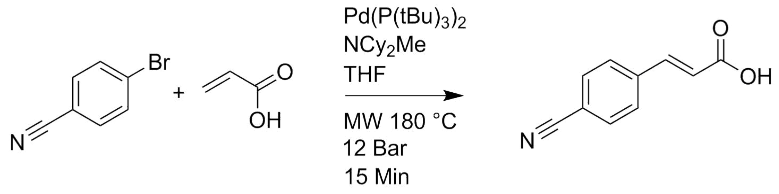

Trans-4-cyanocinnamic acid, [4-CN-CAH] Synthesis. A 20 mL Biotage microwave reaction vial equipped with a teflon magnetic stirbar was charged with 4-bromobenzonitrile (10 mmol), acrylic acid (11 mmol), N,N-dicyclohexylmethylamine (11 mmol), bis(tri-tert-butylphosphine)palladium (1.0 mol %) and THF (10 mL). The vial was capped and the mixture irradiated with a ceiling temperature of 180°C for 15 minutes. The crude product was then extracted three times with 200 mL dichloromethane and 200 mL acidic aqueous (5% vol HCl) solution. The organic layer was dried with $MgSO_4$ and the solvent removed using a rotary evaporator. The crude product was further purified by recrystallization in ethanol.

The product was purified by recrystallization from ethanol affording white needle like crystals (92% yield). The product was sufficiently pure after this step to use directly as a ligand for the oleate exchange process.

^{1}H NMR (500 MHz, DMSO-d_6) δ 12.63 (s, 1H), 7.89 – 7.81 (m, 4H), 7.60 (d, *J* = 15.9 Hz, 1H), 6.67 (d, *J* = 15.9 Hz, 1H).
^{13}C NMR (126 MHz, DMSO-d_6) δ 167.70, 142.46, 139.35, 133.25, 123.34, 119.15, 112.61.

Supplementary Note 2.

Computation methods for functionalized cinnamic acid ligand calculations. The functionalized cinnamic acid ligands were optimized in the trans conformation *i.e.*, the acidic proton bound on either one of the carboxylic oxygen atoms. We computed the ligand dipole moment as projected on the molecular axis. The molecular axis was defined using atoms in position 1 and 4 of the benzene ring. It is known that dipole moments converge slowly as a function of the volume of the cell, we thus performed additional tests to verify the convergence of the results. We found it necessary to use cells as large as two times the greatest distance between two atoms within each model plus 5 Å.

Supplementary Note 3.

XPS Data Analysis. The valence band maximum with respect to Fermi energy ($\mathrm{E_F} - E_{\mathrm{VBM}}$) (Fig. 3b) is not a simple extraction from the rise in photoelectron intensity (($E_{\mathrm{F}} - E_{\mathrm{onset}}$) is the intersection of the linear extraction of the rise in intensity and the baseline). Due to a low density of states at the valence band maximum, a correction to the extracted onset of photoelectron intensity is needed to determine the ($E_{\mathrm{F}} - E_{\mathrm{VBM}}$) value for PbS QD films. This has been previously discussed by Miller *et*

al.[2] Briefly, the correction depends on the band gap (E_g = optical band gap + exciton binding energy); the correction to the onset of photoelectron intensity is *correction = 0.382 - 0.226(E_g).* For this study, the optical band gap and exciton binding energy of the native PbS QD ligand is 1.27 and 0.09 eV, respectively, which gives E_g = 1.36 eV and a correction of 0.075 eV. Therefore, $(E_\mathrm{F} - E_\mathrm{VBM})$ = $(E_\mathrm{F} - E_\mathrm{onset})$ – (0.075 eV) for the PbS QD films used in this study.

The XPS data highlights the influence of the ligand dipole. Fig. 3b and Supplementary Table 2 shows that the measured $E_\mathrm{F} - E_\mathrm{VBM}$ value is similar for all of our R-CA$^-$ ligand exchanges. This strongly suggests that the attachment of the ligand to the surface is the same for all ligands and that the ligand minimally perturbs the Fermi level within the PbS band gap. The significant change is observed in the XPS secondary electron cut-off region (Fig. 3a), which is tuned by the ligand dipole.

We are able to extract meaningful XPS data from QD films made from the as-synthesized OA$^-$/PbS QDs (Fig. 3, black traces). In our lab, previous XPS measurements of OA$^-$/PbS QD films where the QDs were synthesized using other methods (Hines and Scholes / $(TMS)_2Si$ / *in-situ* generated $Pb(OA^-)_2$) showed high levels of photocharging, making all of the data collected subject to scrutiny and the band positions of OA$^-$/PbS QDs difficult to determine. In fact, the majority of XPS literature data are for QD films fabricated using layer-by-layer ligand exchange techniques so that the resulting films are electronically conductive. We postulate that the narrow size distributions and pristine surface of the Owen thiourea PbS QD synthesis allows for improved charge transport, and subsequently, low levels of film photocharging (verified *via* power dependence of the secondary electron cutoff).

XPS surface-sensitive elemental analysis is shown in Supplementary Table 2. Chlorine from solvent mixtures was not detected, suggesting removal of any residual solvent through film annealing and exposure to high vacuum.

Supplementary Note 4.

Functionalized Benzenethiolate Ligand Exchange. We performed a near-complete solution-phase ligand exchange using [4-CH_3-S$^-$][TEA$^+$] following a similar exchange protocol as that developed by Giansante *et al.*[3] The FT-IR spectra of the QDs pre- and post-exchange, as well as the neat ligands, can be seen in Supplementary Fig. 1. We attempted to adapt the exchange procedure for [4-CF_3-S$^-$][TEA$^+$], [4-NH_2-S$^-$][TEA$^+$], and [4-H-S$^-$][TEA$^+$]; however, after a single step of PCR purification, the ligand exchanged QDs irreversibly aggregated and we were unable to re-disperse the exchanged ligand/QD complexes in any combination of solvents/solvent mixtures on hand. We deposited films of the solution-phase ligand exchanged 4CH_3-S$^-$/QDs *via* a single spin coating deposition step for XPS measurements.

Since we were unable to perform solution-phase exchanges using other 4-R-SH ligands, we fabricated electronically conductive films of 4-R-S$^-$/QDs using solid-state, layer-by-layer (LbL) film fabrication techniques using sequential spin coating cycles. We depositing a thin layer of OA$^-$/QDs from octane, soaked the film in a 1 mM [4R-S$^-$][TEA$^+$] ACN solution, and washed the film twice with neat hexane and ACN. Ligands used for this process included [4CH_3-S$^-$][TEA$^+$] and [4H-S$^-$][TEA$^+$] - LbL film fabrication using [4-CF_3-S$^-$][TEA$^+$] and [4-NH_2-S$^-$][TEA$^+$] were unsuccessful. Raw XPS spectra, XPS band edge energies, XPS

elemental analysis results, and calculated ligand dipoles for these films are presented in Supplementary Fig. 2 and Supplementary Table 3.

Supplementary Note 5.

Functionalized CA^- Capped PbS QD Models. We built charge neutral QD models with Pb excess[4,5] and by satisfying charge orbital balance:[6,7] the formal charge of the PbS QD core is balanced by the formal and opposite charge of the R-CA^- ligands bound on the surface of the QD. We started by cutting out isolated cubes from bulk PbS. Cubes with odd number of layers were off-stoichiometric while cubes with even number of layers were stoichiometric. We generated three structural models with varying amount of ligands. For model A, with formula $[Pb_{43}S_{38}][R\text{-}CA^-]_{10}$, we first generated a five layer cube and cut off atoms to define a QD with small (111) and (110) facets and a near spherical shape. The surface was then passivated with 10 R-CA^- ligands that were bound both on (111) and (100)-like facets. The ligands coordinated the surface Pb atoms in a mixture of chelating and bridging conformations. Model B, with formula $[Pb_{62}S_{55}I_8][R\text{-}CA^-]_6$, was constructed by removing the eight corner atoms of a QD with six layers and attaching six R-CA^- ligands to the (100) mini-facets in a bridging configuration and also passivating the corners with iodine atoms to keep charge-orbital balance. Model C, formula $[Pb_{44}S_{40}I_4][R\text{-}CA^-]_4$, was made by removing selected atoms from the PbS core of model B in a way to have larger (111) facets. The surface was then passivated with four R-CA^- ligands while making sure that all the ligands align along one Cartesian direction. We made this choice for computational reasons: by having ligands point in only one direction, we only had to use larger cell size in one Cartesian direction. To ensure charge balance, four iodine atoms were used to passivate the remaining four (111) facets. In all three cases, we generated several different binding conformations, and we verified that the models discussed here are the most stable structures. Supplementary Fig. 3 shows the ball-and-stick structural model of the relaxed 4-H-CA^- covered models. The large variety of these models sampling several possible shapes, facets, and binding moieties allowed us to draw robust conclusions in regards of the band edge position and optical absorption of the ligand/QD complexes.

Supplementary Note 6.

Computation methods for ligand/QD band edge shifting calculations. The band edge shift of the QDs was computed by measuring the HOMO and LUMO energy of the ligand/QD complexes on an absolute energy scale with respect to the vacuum level as a function of the aromatic functional group of the R-CA^- ligands. The HOMO was always localized on the QD core, with the exception of 4-$N(CH_3)_2$-CA^-, where the ligand states were too high in energy relative to the energy of QD states, and thus the HOMO was localized on the ligands. This is likely a result of the underestimation of ionization potentials using density functional theory in the PBE approximation. In order to disentangle this effect from the ligand dipole induced shift of the QD states, we performed a projected density of states analysis and defined the QD HOMO/LUMO as the first state from the band edge that was localized on the QD core. The projected density of states DOS_Ω for atoms *i* belonging to region Ω is defined as:

$$\mathrm{DOS}_\Omega(\epsilon) = \sum_j \sum_{i\in\Omega} \left|\langle\varphi_i|\psi_j\rangle\right|^2 \delta(\epsilon_j - \epsilon), \qquad (1)$$

where φ_i are atomic orbitals and ψ_j are Kohn-Sham states with energy ε_j. We defined two regions: one that is comprised of the PbS QD core ($\mathrm{DOS}_{\mathrm{QD}}(\epsilon)$) and another one that contains the ligands ($\mathrm{DOS}_{\mathrm{ligand}}(\epsilon)$). See Supplementary Fig. 4a for a breakdown of these contributions for ligand/QD complex model C.

Using the above definition of the ligand/QD complex HOMO/LUMO states, the computed band edge shifts for the various R-CA$^-$/QD models are shown in Supplementary Fig. 4b as a function of the projected dipole moment of the R-CAHs.

Supplementary Note 7.

Polarizability effects on ligand/QD band edge shifting. Although the predicted range of band edge shift was in good agreement with the experimental results, there were a few cases where the agreement was not perfect. Thus, as summarized in the main manuscript, we have also considered additional effects that may be responsible for the residual differences.

We used computational methods to study ligand interactions on the surface of QDs (see below for intra-QD ligand shell effects and the main text for inter-QD ligand shell effects). Although surface coverage effects are taken into account by our electrostatic model, early experiments on monolayers of self-assembled molecules on bulk CdSe films[8] suggested that in the case of close packed ligand coverage, the effective dipole felt by the surface might be reduced due to depolarization effects. Since depolarization effects are proportional to the polarizability the ligand, and fluorinated bonds are known to be less polarizable than other considered functional groups (see below for a longer discussion on the polarizabilities of the ligands), we anticipated that the residual differences between theory and experiments might be attributed to functional group dependent depolarization effects.

In our simulations on isolated QDs, we did not use full surface coverage to reduce the computational burden of the calculations, however, we studied full surface coverage in the case of slabs. We thus analyzed depolarization effects on flat surfaces, where the shift in the band edge energy is given by:

$$\Delta\epsilon \propto \tilde{p}/\varepsilon A. \quad (2)$$

Here, ε incorporates dielectric screening effects at the monolayer/slab interface and A is the surface area. Since the dielectric constant is not well defined for a monolayer of ligands, following earlier works,[9,10] we have developed a classical electrostatic approach to take into account dielectric screening, sometimes also called local field effects. In this picture, the dipole moment of a ligand is renormalized due to the electric field of neighboring dipoles. The effective surface dipole is reduced and the band edge shift becomes:

$$\Delta\epsilon \propto \tilde{p}/(1 + C\frac{\alpha}{a^3})A, \quad (3)$$

where α is the polarizability of the ligands, a is the lattice constant of the monolayer and C is a unitless constant that is dependent on the geometry of the 2D lattice of the monolayer. It is 9.03 for a square and 11.04 for a hexagonal lattice,[11] the latter is relevant for bulk PbS(111) surfaces. Additional effects,

(4)

such as screening from the substrate and the presence of image dipoles, should also be taken into account. Indeed, according to Ref. [9], the presence of a substrate with dielectric constant ε_m further contributes to the depolarization of the ligand dipoles:

$$\Delta\epsilon \propto \tilde{p}/(1 + C\frac{\alpha}{a^3}\frac{2\varepsilon_m}{\varepsilon_m + 1})A$$

Since the dielectric constant of PbS is high (for example, the low frequency dielectric constant is 169),[12] image dipole effects are changing the proportionality constant of the polarizability by a factor of two to a very good approximation.

In order to test the above electrostatic model, we computed the polarizabilities of the ligands using a finite field approach. A small external sawtooth potential was applied in each Cartesian direction j and we measured the change in the dipole moment along Cartesian direction i. The polarizability tensor α_{ij} is then obtained by a central difference formula:

$$\alpha_{ij} = \frac{p_i(\Delta E_j) - p_i(-\Delta E_j)}{2\Delta E_j}. \qquad (5)$$

We used an electric field with magnitude of 0.001 Hartree atomic units. We only allowed the charge density to respond to the external potential (the atoms were not allowed to relax), therefore the computed values are high frequency polarizabilities and are expected to underestimate the full, static polarizabilities. We also computed the polarizabilities projected on the molecular axis v:

$$\alpha = v_i \alpha_{ij} v_j. \qquad (6)$$

We also verified on a single ligand that the finite field calculations are in the linear regime by computing the dielectric constant of the ligand in a cubic box with lattice constant of 40 Å using density functional perturbation theory techniques and then using the Clausius-Mossotti formula to extract the polarizability. The isotropic polarizabilities computed with the two different techniques agreed within 2% for 4-H-CAH. All finite field polarizability calculations were run in a cell size of at least 25 Å and we verified the convergence of the results by increasing the box size of the 4-H-CAH by 5 Å and finding that the results only changed by 0.1%. Supplementary Fig. 6a shows the polarizability and projected polarizabilities of the ligands as a function of the functional group on the ligands. We find that the polarizability is proportional to the HOMO-LUMO gap of the molecules. More importantly, we see that the polarizability of the 4-CN-CAH ligand is only marginally larger than those of 3,5-F-CAH and 4-CF_3-CAH and thus it is not likely that depolarization are alone responsible for the observed differences. This conclusion is also supported by Supplementary Fig. 6b, which represents the renormalized projected dipoles (p') using the projected polarizabilities: $p' = p/(1 + C\frac{\alpha}{a^3}2)$: the absolute magnitude of the renormalized dipoles change substantially, but the trend did not change. Here, we used $C = 11.04$ and $a = 4.25$ Å, which are the lattice sum and the lattice constant of the surface unit cell of the PbS(111) surface, respectively.

Although the above analysis suggests that depolarization effects are very likely not responsible for the observed differences in the band edge energies, we constructed slabs of bulk PbS (111) with two different surface coverages to further analyze band edge shifts using more realistic models. Slab (A) was obtained by making a Pb terminated 3x1 (111) slab of PbS with inversion symmetry and then removing one out of three Pb atoms on both facets while maintaining the inversion symmetry. The formula of the slab was $Pb_{13}S_{12}$. Both facets were passivated with deprotonated cinnamic acids bound in the groves of the top Pb layers. We chose this binding conformation because recent calculations on simple acids suggest that this is the most energetically favorable binding conformation under similar surface coverage conditions.[13] The surface coverage was ~44 $Å^2$/ligand. Slab (B) had a 1x1 surface unit cell of the (111) slab of bulk PbS again with inversion symmetry. The formula was Pb_5S_4. We then passivated both facets with deprotonated cinnamic acids. We tried several different ligand arrangements and found that the chelating binding is the most energetically favorable. We used a k-point sampling of at least 4x4x1 of the surface primitive unit cell, which is sufficient to obtain converged trends.

We then computed the valence band maximum (VBM) by measuring the VBM of the slabs relative to the average electrostatic potential in the middle of the vacuum region along the direction z. By construction, the dipole moment of the entire slab was zero and thus the electrostatic potential was flat in the vacuum region. We note that these slabs are not thick enough and thus confinement effects can be observed for the absolute position of the VBM and for the band gap. However, the relative trends between the different ligands should be largely unaffected. Supplementary Fig. 7(a-d) shows the structural models of both slabs and Supplementary Fig. 7e represents the VBM w.r.t vacuum as a function of the functional group on the ligands. Since we were interested only in ligands that were either fluorinated or were outliers in the experiment, we did not consider the ligands with larger functional groups. Interestingly, the data shows that depolarization effects may indeed take place, since the ratio of the ranges of band edge shift for the two different models is about 1.8. Should depolarization effects not take place, this value would equal 3.0, since the surface coverage differs exactly by three. However, the trend still follows the trend predicted by the dipoles, in agreement with the analysis above about the surface polarization effects. We thus conclude that surface polarization is unlikely to be the only property responsible for the observed differences.

Supplementary References

1. Jeong, K. S., Deng, Z., Keuleyan, S., Liu, H. & Guyot-Sionnest, P. Air-Stable n-Doped Colloidal HgS Quantum Dots. *J. Phys. Chem. Lett.* **5,** 1139–1143 (2014).
2. Miller, E. M. *et al.* Revisiting the Valence and Conduction Band Size Dependence of PbS Quantum Dot Thin Films. *ACS Nano* **10,** 3302–3311 (2016).
3. Giansante, C. *et al.* Colloidal Arenethiolate-Capped PbS Quantum Dots: Optoelectronic Properties, Self-Assembly, and Application in Solution-Cast Photovoltaics. *J. Phys. Chem. C* **117,** 13305–13317 (2013).
4. Moreels, I. *et al.* Composition and Size-Dependent Extinction Coefficient of Colloidal PbSe Quantum Dots. *Chem. Mater.* **19,** 6101–6106 (2007).
5. Moreels, I., Fritzinger, B., Martins, J. C. & Hens, Z. Surface Chemistry of Colloidal PbSe Nanocrystals. *J. Am. Chem. Soc.* **130,** 15081–15086 (2008).
6. Frenzel, J., Joswig, J.-O. & Seifert, G. Optical Excitations in Cadmium Sulfide Nanoparticles. *J. Phys. Chem. C* **111,** 10761–10770 (2007).
7. Voznyy, O. *et al.* A Charge-Orbital Balance Picture of Doping in Colloidal Quantum Dot Solids. *ACS Nano* **6,** 8448–8455 (2012).
8. Bruening, M. *et al.* Polar Ligand Adsorption Controls Semiconductor Surface Potentials. *J. Am. Chem. Soc.* **116,** 2972–2977 (1994).
9. Iwamoto, M., Mizutani, Y. & Sugimura, A. Calculation of the dielectric constant of monolayer films on a material surface. *Phys. Rev. B* **54,** 8186–8190 (1996).
10. Macdonald, J. R. & Jr, C. A. B. Work Function Change on Monolayer Adsorption. *J. Chem. Phys.* **39,** 412–422 (1963).
11. Topping, J. On the Mutual Potential Energy of a Plane Network of Doublets. *Proc. R. Soc. Lond. Math. Phys. Eng. Sci.* **114,** 67–72 (1927).
12. Madelung, O., Rossler, U. & Schulz, M. *Non-Tetrahedrally Bonded Elements and Binary Compounds I*. (Springer, 1998).
13. Zherebetskyy, D. *et al.* Hydroxylation of the surface of PbS nanocrystals passivated with oleic acid. *Science* **344,** 1380–1384 (2014).
